# Perceptual encoding benefit of visual memorability on visual memory formation

**DOI:** 10.1016/j.cognition.2024.105810

**Published:** 2024-05-11

**Authors:** Chaoxiong Ye, Lijing Guo, Nathan Wang, Qiang Liu, Weizhen Xie

**Affiliations:** a Institute of Brain and Psychological Sciences, Sichuan Normal University, Chengdu 610066, China; b Department of Psychology, University of Jyväskylä, Jyväskylä 40014, Finland; c School of Education, Anyang Normal University, Anyang 455000, China; d Johns Hopkins University, Baltimore, MD 21218, United States of America; e Department of Psychology, University of Maryland, College Park, MD 20742, United States of America

**Keywords:** Memorability, Perception, Visual short-term memory, Visual long-term memory

## Abstract

Human observers often exhibit remarkable consistency in remembering specific visual details, such as certain face images. This phenomenon is commonly attributed to visual memorability, a collection of stimulus attributes that enhance the long-term retention of visual information. However, the exact contributions of visual memorability to visual memory formation remain elusive as these effects could emerge anywhere from early perceptual encoding to post-perceptual memory consolidation processes. To clarify this, we tested three key predictions from the hypothesis that visual memorability facilitates early perceptual encoding that supports the formation of visual short-term memory (VSTM) and the retention of visual long-term memory (VLTM). First, we examined whether memorability benefits in VSTM encoding manifest early, even within the constraints of a brief stimulus presentation (100–200 ms; Experiment 1). We achieved this by manipulating stimulus presentation duration in a VSTM change detection task using face images with high- or low-memorability while ensuring they were equally familiar to the participants. Second, we assessed whether this early memorability benefit increases the likelihood of VSTM retention, even with post-stimulus masking designed to interrupt post-perceptual VSTM consolidation processes (Experiment 2). Last, we investigated the durability of memorability benefits by manipulating memory retention intervals from seconds to 24 h (Experiment 3). Across experiments, our data suggest that visual memorability has an early impact on VSTM formation, persisting across variable retention intervals and predicting subsequent VLTM overnight. Combined, these findings highlight that visual memorability enhances visual memory within 100–200 ms following stimulus onset, resulting in robust memory traces resistant to post-perceptual interruption and long-term forgetting.

## 1. Introduction

In spite of large individual differences in people’s past experiences and memory capacity (Unsworth, 2019), certain aspects of everyday life consistently leave a lasting impression on us. For example, we may form a robust memory of a person with a mere glimpse of a face, while many other individuals we encounter in our daily lives are inevitably forgotten (Bainbridge, Isola, & Oliva, 2013). This phenomenon can be partly attributed to the variability in the memorability of visual stimuli in our environment, defined as the likelihood of an item triggering visual recognition across people (Bainbridge, 2019; Salthouse, 2017). Although the exact reasons behind why certain visual stimuli are more memorable than others remain unclear (Rust & Mehrpour, 2020), the consistency of this phenomenon across large-scale populations has rekindled a historical interest in this puzzle (Rubin, 1985).

In the visual domain, memorability has been considered a stimulus attribute that is independent of an observer’s past experiences and specific task contexts (see a recent review in Bainbridge, 2019). This idea finds support in the predictability of visual memorability using feedforward deep neural network (DNN) models trained solely on pixel-level information of a visual image (Jaegle et al., 2019; Khosla, Raju, Torralba, & Oliva, 2015). Remarkably, these stimulus-driven models not only accurately predict how memorable an image is to human observers, but also capture neuronal responses in the inferotemporal cortex in monkeys despite the animals’ limited prior knowledge about the image content (e.g., cars and fire hydrants, Jaegle et al., 2019). These findings therefore underscore the notion that the processes encompassing semantic or associative knowledge might not adequately account for the construct of visual memorability despite their well-recognized impact on people’s overall memory likelihood (Aka, Bhatia, & McCoy, 2023; Kennet, McGuire, Willis, & Schaie, 2000; Madan, 2021; Xie, Bainbridge, Inati, Baker, & Zaghloul, 2020). Consequently, this understanding has prompted the hypothesis that the advantage conferred by visual memorability potentially emerges from enhanced cognitive processing during the initial stages of perceptual encoding as opposed to subsequent post-perceptual processes (Bainbridge, 2019; Lin, Yousif, Chun, & Scholl, 2021; Mohsenzadeh, Mullin, Oliva, & Pantazis, 2019).

Supporting this notion, previous research has attempted to reveal how quickly and automatically this advantage in visual memorability emerges. For example, using a rapid serial visual presentation (RSVP) task, Broers, Potter, and Nieuwenstein (2018) found that high memorability images were better detected relative to low memorability ones, even with extremely brief presentation duration (e.g., 13 ms). The rapid processing of memorability has also been demonstrated in studies using neuroscience techniques. For instance, by employing the high temporal resolution of magnetoencephalography (MEG) during an RSVP task, Mohsenzadeh et al. (2019) tracked the temporal neural signature of memorability across the brain, pinpointing the timing of memorability decoding at approximately 150–230 ms after stimulus onset. These results unveiled a relatively early and sustained signal associated with memorability. However, while findings in RSVP settings have underscored the rapid effects of visual memorability on perceptual processing (as short as 13 ms), it remains uncertain the extent to which these short-term perceptual enhancements can directly explain the enduring memory advantages conferred by visual memorability. Moreover, because individual perceptual features such as color, contrast, or spatial frequency cannot reliably predict the memorability of a visual stimulus (Bainbridge, 2019; Bylinskii, Isola, Bainbridge, Torralba, & Oliva, 2015; Isola, Xiao, Torralba, & Oliva, 2011), direct behavioral evidence supporting the perceptual encoding benefit hypothesis has remained limited, especially when we consider the following additional challenges.

First, as previous studies demonstrating visual memorability benefits in visual memory often relied on relatively longer or less controlled encoding durations in an online setting (e.g., 1–2 s; Bainbridge, 2019; Bylinskii et al., 2015; Gillies et al., 2023; Isola et al., 2011; Lin et al., 2021), it remains unclear whether these memorability benefits in visual memory would still emerge within a shorter perceptual encoding time such as around 100 to 200 ms (Thorpe, Fize, & Marlot, 1996). Conceptually, the benefits of memorability that emerge during prolonged encoding times may not solely be attributed to early perceptual encoding advantages. Post-perceptual processes, such as semantic associations or other more elaborative encoding mechanisms (Craik & Lockhart, 1972), could also play a role. Second, these previous studies have rarely experimentally intervened in post-perceptual processes, such as the post-stimulus consolidation process that transfer fragile sensory input into durable visual short-term memory (VSTM) (Becker, Miller, & Liu, 2012; Ricker & Cowan, 2014; Ricker, Nieuwenstein, Bayliss, & Barrouillet, 2018; Vogel, Woodman, & Luck, 2006; Xie, Lu Sing, Martinez-Flores, & Zhang, 2022; Xie & Zhang, 2017c). It remains unknown whether the memorability benefits observed during visual memory formation result from the probabilistic nature of perceptual encoding or if they affect post-perceptual consolidation process in a time dependent manner. For example, past research has suggested that VSTM consolidation is influenced by participants’ visual familiarity with, or prior knowledge of, a given visual stimulus (Blalock, 2015; Xie & Zhang, 2017c, 2018). Such a visual familiarity effect may reflect the impact of pre-existing visual long-term memory (VLTM) on VSTM formation, in which familiar items may be consolidated into VSTM more rapidly than less familiar ones (Xie & Zhang, 2022). It remains plausible that visual memorability might exert its influence on VSTM formation through a similar post-perceptual VSTM consolidation process. Third, while memorability estimates seems to be stable across retention intervals (Goetschalckx, Moors, & Wagemans, 2018), given past mixed findings regarding the relationship between the memorability benefit in VSTM and that in VLTM (e.g., Gillies et al., 2023; also see introduction in Experiment 3), it remains unclear the extent to which the early perceptual encoding benefit introduced by visual memorability can account for the subsequent increase in memory likelihood across various retention intervals, extending beyond the timescale in a continuous recognition task setting (Lin et al., 2021).

To fill these gaps, this study therefore aims to examine how visual memorability influences visual memory formation across VSTM and VLTM. In particular, we seek to understand whether visual memorability leads to a perceptual encoding benefit or accelerates VSTM consolidation speed and whether these visual memorability benefits in VSTM can directly account for a subsequent VLTM boost – two key issues that have not been clarified by the past research (Broers et al., 2018; Mohsenzadeh et al., 2019). Addressing these issues is helpful for gaining insights into the cognitive processes underlying the memorability benefits in visual memory tasks. Furthermore, clarifying the extent to which successes or failures in remembering typically memorable visual information can be ascribed to a perceptual or memory consolidation limitation may also have translational implications in aging and clinical contexts. Hence, the main goal of the current study is to assess the degree to which perceptual encoding benefit can explain memorability-related improvement in task performance observed across VSTM and VLTM tasks, with a specific focus on the following critical predictions.

### 1.1. Perceptual encoding benefits of visual memorability should emerge early in VSTM

First, if visual memorability indeed enhances visual memory formation during perceptual encoding rather than the post-perceptual VSTM consolidation process, this advantage should emerge early. While previous studies on visual memorability have primarily focused on tasks and findings in the field of VLTM (Bainbridge, 2019; Rust & Mehrpour, 2020), recent research has also observed enhanced performance in VSTM tasks when presented with memorable visual stimuli (Gillies et al., 2023). Nevertheless, these studies have not examined factors such as the duration of stimulus presentation that might influence task demands on early perceptual processing. This omission leaves us uncertain about whether visual memorability has an early or a later impact during VSTM formation.

Previous studies have demonstrated that the duration of stimulus presentation can interact with stimulus features and task contexts to influence the transfer of an individual item from fragile sensory inputs to stable VSTM representations (Becker et al., 2012; Hao, Becker, Ye, Liu, & Liu, 2018; Long, Ye, Li, Tian, & Liu, 2020; Ye et al., 2017, 2019; Ye et al., 2020). For example, according to a two-phase model of VSTM encoding (Ye et al., 2017), bottom-up stimulus-driven factors tend to exert an early impact on VSTM encoding (i.e., early phase) before the involvement of top-down control-related mechanisms (i.e., late phase). At the behavioral level, these effects can be detected under experimental conditions using different stimulus presentation durations in a VSTM task. For example, early perceptual encoding benefits observed due to stimulus-driven factors often emerge early in a condition with a stimulus presentation duration at the perceptual threshold (< 150 ms) and can carry over into conditions with a longer stimulus encoding time (Hao et al., 2018). In contrast, the effect of post-perceptual processes, such as voluntary control on VSTM encoding, often becomes obvious only when a sufficient stimulus presentation duration is given, typically exceeding several hundred milliseconds (Long et al., 2020). If visual memorability benefits in VSTM task performance emerge even with a brief stimulus presentation duration (e.g., 100 ms), it is likely that such an effect may be attributed to the early perceptual encoding stage prior to post-perceptual processes, such as voluntary control or elaborative encoding. However, if the visual memorability benefits for a VSTM task only emerge with longer stimulus presentation durations, this would pose a challenge to the early perceptual encoding benefit hypothesis. To test these possibilities, Experiment 1 manipulated the presentation duration of VSTM study items to investigate the effects of visual memorability on VSTM formation.

### 1.2. Perceptual encoding benefits of visual memorability should be resilient to post-stimulus masking

Second, considering the probabilistic nature of visual perception (Jabar & Fougnie, 2022), a stimulus may be perceived as a sample from a distribution of internal representations with a certain degree of likelihood (Xie & Zhang, 2023b). Given a fixed stimulus presentation duration, if a percept is more advantageously formed during this time, a corresponding benefit should be evident in the likelihood of encoding that percept into VSTM during the subsequent consolidation period (Xie & Zhang, 2017c). This early perceptual encoding benefit is conceptually distinct from changes in the speed of VSTM consolidation driven by participants’ pre-existing VLTM of or familiarity with the task content. In the latter case, the likelihood of an item being encoded into VSTM exhibits a time-dependent pattern after stimulus presentation, depending on how familiar an observer is with the task content (Blalock, 2015; Ngiam, Khaw, Holcombe, & Goodbourn, 2019; Xie & Zhang, 2017c, 2018). These predictions are consistent with previous theories on visual processing, wherein the consolidation of VSTM is hypothesized to be constrained by both an initial perceptual intercept (i.e., the time it takes to form a percept) and consolidation speed, presumably within a VSTM storage limit (Bundesen, 1990).

Previous research has developed a masking procedure to reveal these effects on VSTM formation (Enns & Di Lollo, 2000), by inserting random pattern masks between memory and mask arrays at different SOAs (Stimulus Onset Asynchronies). For example, using this paradigm, previous research has demonstrated that participants’ pre-existing VLTM of or familiarity with task stimuli primarily modulates the speed of VSTM consolidation, resulting in a higher likelihood of items being encoded into VSTM as the memory-and-mask SOA increases within a VSTM storage capacity limit (Xie & Zhang, 2017c, 2022) – a finding later supported by subsequent event-related potential (ERP) evidence (Xie & Zhang, 2018). These familiarity effects are in stark contrast to the predictions from a perceptual encoding benefit, as the early perceptual encoding benefit driven by stimulus properties may primarily shorten the time it takes to form a percept (i.e., perceptual intercept in Bundesen, 1990), without altering the subsequent VSTM consolidation process. Given the same stimulus presentation duration, this should manifest as a constant boost in task performance across memory-and-mask SOAs (Xie et al., 2022; Xie & Zhang, 2017c). To examine whether the effects of visual memorability on VSTM formation are similar or different from these familiarity effects, using a similar experimental design (Ngiam et al., 2019; Xie et al., 2022; Xie & Zhang, 2017c), Experiment 2 investigated participants’ VSTM for high- and low-memorability images across different memory-and-mask SOAs while controlling for the presentation duration of VSTM study items.

### 1.3. Perceptual encoding benefits of visual memorability should manifest across retention timescales

Finally, the early perceptual benefits observed for memorable stimuli in VSTM are expected to contribute to the subsequent formation of robust VLTM. Given that VSTM serves as a gatekeeper for VLTM formation (Fukuda & Vogel, 2019), the strong VSTM representations resulting from the process at the early perceptual encoding stage should facilitate the subsequent formation of VLTM. This prediction suggests that VSTM task performance driven by the early perceptual encoding benefits of memorable stimuli may account for a significant amount of variance in the subsequent performance in VLTM tasks involving these stimuli. This perceptual encoding benefit account provides a parsimonious explanation for how visual memorability enhances visual memory formation over time. However, it is also possible that VLTM formation is influenced by additional factors, such as interference (Underwood, 1957; Xie, Park, Zaghloul, & Zhang, 2020). Therefore, if the participants do not consistently consolidate the visual information of a VSTM task into VLTM, the benefits observed during VSTM due to memorability may not directly translate into improved VLTM performance. To distinguish between these possibilities and reveal the memorability benefits on visual memory across short and long delays, we conducted Experiment 3, in which participants completed a surprise visual recognition test on the second day using stimuli with varying levels of memorability after engaging in a VSTM task with different delay intervals.

### 1.4. Preview of the current study

The current study assessed participants’ performance in VSTM and VLTM tasks using memorable and forgettable face photographs selected from an established stimulus database (Bainbridge et al., 2013) to test three key predictions from the perceptual encoding benefit hypothesis of visual memorability on visual memory formation. Prior to the main experiments, we conducted a Pilot experiment to ensure that the chosen stimuli varied in their memorability levels in our study population but were matched in participants’ familiarity and various multi-dimensional attributes that capture the appearance of these face images (e.g., race, gender, etc.). Afterward, we proceeded to examine participants’ performance in a VSTM change detection task using the selected face images of either high or low memorability. We manipulated the duration allowed for perceptual encoding in two ways: by varying the encoding duration itself (Experiment 1) and by introducing post-stimulus masking at different memory-and-mask SOA (Experiment 2). In both experiments, we consistently found that participants showed better VSTM change detection task performance for memorable relative to forgettable faces, even when the perceptual encoding duration was as brief as 100–200 ms. Moreover, in Experiment 3, we investigated how the memorability benefit arising from early perceptual encoding evolves over time by manipulating the retention interval from a few seconds (1.4 s, 4 s, and 10 s) to 24 h after the initial VSTM task. Remarkably, we find that the memorability benefit observed in VSTM persists for up to 24 h, predicting VLTM task performance within participants. Collectively, our data systematically reveal the robust perceptual encoding benefit conferred by visual memorability on visual memory formation, highlighting its distinctiveness from that conferred by participants’ familiarity with the task content and its enduring impact on VSTM and VLTM.

## 2. Pilot experiment

To ensure valid visual memorability estimates for participants from a cultural background that is different from the original research, we chose a set of images from the 10 k US Adult Faces Database (Bainbridge et al., 2013) and confirmed the memorability of these images in the current research sample.

### 2.1. Methods

#### 2.1.1. Participants

Forty Chinese college students (7 males, 33 females, 0 others; mean age 20.03 ± 0.40 [mean ± s.e.m.]) participated in this Pilot experiment, with monetary compensation, at Sichuan Normal University. They self-reported having normal or corrected-to-normal vision. The sample size was determined based on a heuristic that online memorability estimates using ~80 participants could be replicated with data from half of the original sample in a laboratory setting (Xie, Bainbridge, et al., 2020). Therefore, we aimed for a target sample size of 40. As with all other experiments conducted in this study, written informed consent was obtained from participants before the experiment, following the approved protocol set by the ethical committee of Sichuan Normal University (Protocol ID: SCNU-230810).

#### 2.1.2. Materials

We selected a set of face images from the 10 k US Adult Faces Database, comprising 80 high-, 160 medium-, and 80 low-memorability images, adhering to the following criteria. First, all high-memorability face images were selected to have higher memorability estimates (i.e., hit rate ≥ 0.59 in the database) relative to each low-memorability face image (i.e., hit rate ≤ 0.44 in the database). Second, we excluded images featuring faces of famous individuals or celebrities to avoid potential confounds of stimulus familiarity in participants’ task performance (Buttle & Raymond, 2003; Xie & Zhang, 2017c). Third, we ensured that both groups of faces were matched on various key attributes, such as the likelihood of being misremembered (false alarm rate), age, gender, race, image quality, makeup, emotional valence, emotional intensity, attractiveness, friendliness, face direction, and eye direction (see more details in Supplementary Table S1). We then randomly selected 160 faces that do not fall into the high- and low-memorability sets from the original 10 K face data set as filler images (i.e., 0.44 < hit rate < 0.59 in the database). Among these pre-selected face images, the high, medium, and low memorability faces have hit-rate memorability scores of 0.66 ± 0.05, 0.51 ± 0.03, and 0.35 ± 0.06, respectively.

#### 2.1.3. Procedure

**Fig. 1.**
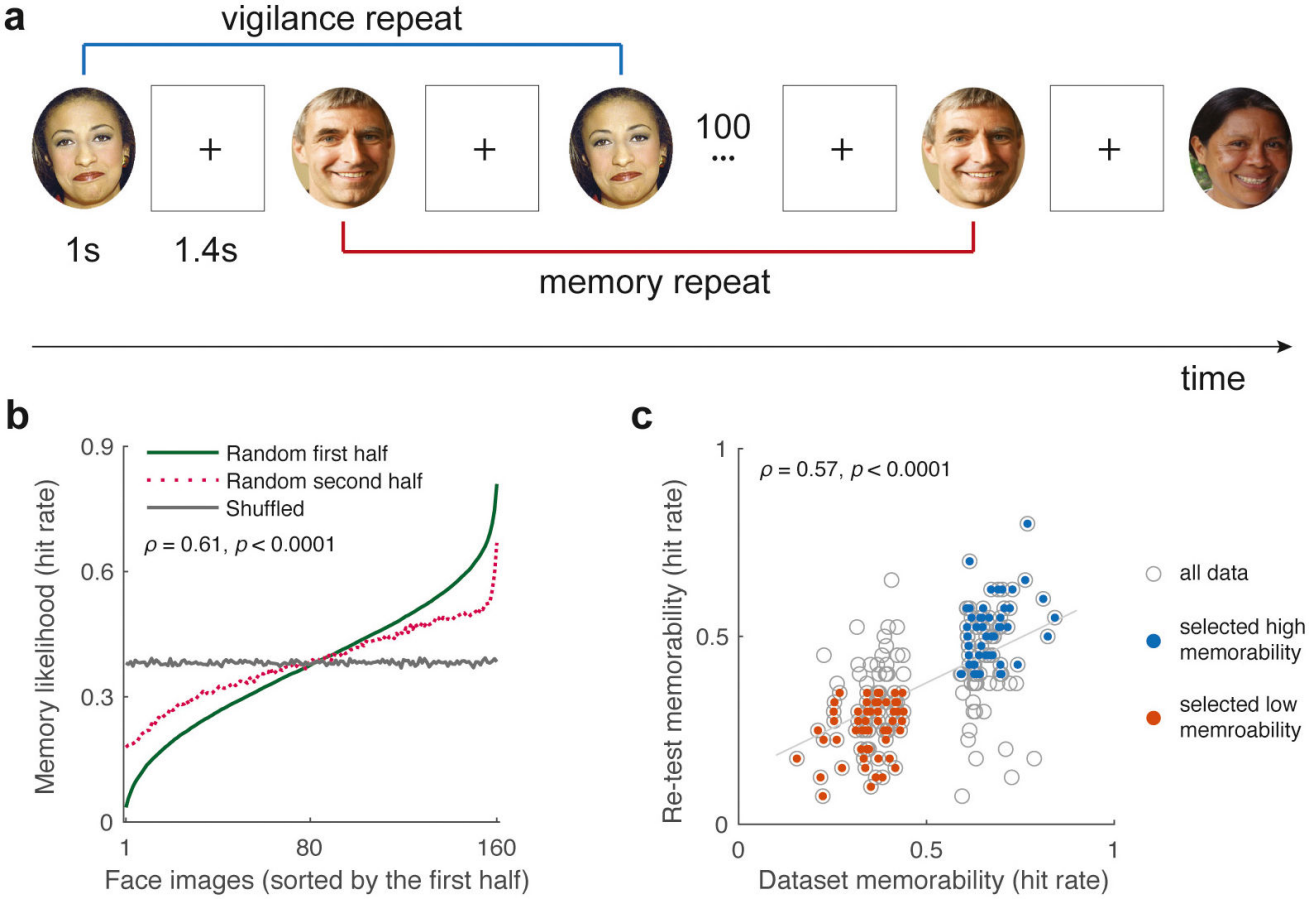
Verifying the memorability of selected face images in the Pilot experiment. (a) Using 320 selected images with varying degrees of visual memorability, the participants completed a continuous visual recognition memory task using the same procedure as previously described (Bainbridge et al., 2013). (b) Split-half analysis of the data indicates that certain face images are reliably remembered by participants across any randomized halves of the current sample. (c) A subset of the selected faces (i.e., solid dots) demonstrated consistent memorability estimates across samples from diverse cultural backgrounds across both Chinese and US participants. Therefore, we selected the subset of images with consistent memorability estimates for subsequent experiments (see Supplementary Table S2 for details in image properties). All face images used in the current figure are under a Creative Commons license for noncommercial use. Details about the copyright information of these face images can be found in the Supplementary Materials.

Participants completed the experiment in a moderately lit laboratory testing room equipped with a 60 Hz LCD monitor positioned at a viewing distance of 60 cm. Each trial involved the presentation of a timed sequence comprising 320 face images, with each face displayed for 1 s followed by an inter-stimulus interval of 1.4 s (see Fig. 1a). Participants were instructed to press the “F” key when a face image was repeated. Target images with either high or low memorability were repeated at intervals of 91–109 images. Images with medium memorability were used as filler images and repeated at intervals of 1–7 images to reduce recognition task difficulty. Memorability measures were obtained as the hit rates for the correct repetition detection of the target face images across participants. We examined whether specific images were consistently remembered or forgotten by the participants using a split-half analysis, as previously described (Xie, Bainbridge, et al., 2020).

### 2.2. Results

Consistent with previous research, we found that participants exhibited a consistent memory likelihood of the target faces, indicated by a high split-half Spearman rank-order correlation between memory likelihood estimates across random halves of the study sample (ρ = 0.61, *p* < 0.001, across 1000 iterations; Fig. 1b). Additionally, our current estimations of memorability, measured as the hit rate of a study item across all participants, showed a strong correlation with the memorability estimates obtained from the original 10 k US Adult Faces Database (ρ = 0.57, p < 0.001; Fig. 1c). These results suggested some robustness and generalizability of visual memorability using face images across different cultural backgrounds. Nevertheless, an important point to note is that variations may occur in subject populations and task procedures, leading to potential discrepancies in memorability estimates at the level of individual images (see scatter plots with gray circles in Fig. 1c). To be conservative, we retained only those images that consistently demonstrated high or low memorability across different populations (see red and blue solid dots in Fig. 1c). As a result, we selected from the database 58 high-memorability face images that were characterized by a hit rate > 0.4 and that exhibited an empirical hit rate of 0.67 ± 0.06. Similarly, we chose 58 low-memorability face images from the database with a hit rate < 0.35 and an empirical hit rate of 0.25 ± 0.06. Importantly, other key attributes of these images, including celebrity status, attractiveness, and affective values, were statistically comparable between the two selected image groups (see Supplementary Table S2).

### 2.3. Discussion

Faces are visual stimuli that carry significant cultural, racial, or ethnic meanings (Goh et al., 2010). As a result, faces perceived as memorable in one cultural context may not necessarily be memorable in another cultural context (Malpass & Kravitz, 1969). However, our data demonstrate that a subset of faces exhibits the same level of memorability across diverse populations, suggesting a certain level of generalizability of these memorability estimates across individuals and contexts.

Importantly, this finding remains robust in our current study, even when the participants are assumed to be unfamiliar with the selected faces. Specifically, as these images were randomly sampled from the 10 k US Adult Faces Database but tested in a research population outside the US, participants are expected to be relatively unfamiliar with these face images. This is confirmed by anecdotal report from the participants, in which they consistently reported that they had not seen these face images before the current experiment. As our participants were drawn from the same college research participant pool, we later on confirmed this by acquiring participants’ subjective ratings of perceived visual familiarity of these faces in subsequent experiments (see Experiments 1 & 2) to unequivocally rule out visual familiarity as a factor in the current research population.

While these data do not exclude the influence from other conceptual or semantic factors on visual memorability, these findings highlight the role of mere stimulus-driven factors in the memorability of the selected face images across cultural contexts and observers. These validation outcomes thus lay the foundation for our subsequent experiments investigating the perceptual impacts of visual memorability on visual memory formation.

## 3. Experiment 1

To examine the first prediction that visual memorability enhances early perceptual processing during VSTM formation, we conducted a VSTM change detection task, manipulating both the presentation duration (100 ms, 200 ms, or 500 ms) and the memorability level (high vs. low) of memory items. If visual memorability, in fact, improves perceptual processing and facilitates VSTM formation, we expect to observe better performance in the VSTM task for items with higher (vs. lower) memorability presented at a shorter presentation duration (e.g., 100 ms and 200 ms). Conversely, observation of a memorability benefit on VSTM task performance only under longer presentation durations (e. g., 200 ms or 500 ms) and not during the shorter presentation duration condition (e.g., 100 ms) would contradict the hypothesis of an early perceptual encoding benefit (Long et al., 2020; Ye et al., 2019). To verify and control for the influence of participants’ familiarity with the task stimuli, we also asked participants to provide ratings of their perceived visual familiarity with the task stimuli (Xie & Zhang, 2017c).

### 3.1. Methods

#### 3.1.1. Participants

A new group of 40 Chinese college participants (6 males, 34 females, 0 others; mean age 19.75 ± 0.29 years) participated in the current experiment with monetary compensation at Sichuan Normal University. This sample size is sufficient to detect a moderate-to-small effect size (e. g., $\eta_{p}^{2}\sim =0.10$) with 80% statistical power at a significance level of 0.05 based on a power analysis (Faul, Erdfelder, Buchner, & Lang, 2009) with a 2 (memorability: high vs. low) × 3 (memory array presentation duration: 100 ms vs. 200 ms vs. 500 ms) design for repeated-measures analysis of variances (ANOVA). All participants reported having normal or corrected-to-normal vision and provided written informed consent prior to participating in the study.

#### 3.1.2. Materials

We used the set of 116 faces with varying levels of memorability (high vs. low) selected in the Pilot experiment. The task involved two consecutive displays: the memory array and the test array. In the memory array, participants were presented with three face images, each measuring approximately 2.26° × 2.61° of visual angle. These images were randomly displayed in one of three locations, equidistantly spaced along an invisible circle with a visual angle radius of 2.9°. During half of the trials, the study faces were randomly selected from the set of highly memorable images, while in the remaining half, the study faces were randomly chosen from the low-memorability images.

The test array consisted of one face image and two placeholders positioned at the original locations of the memory items. The face image presented in the test array could either match the corresponding study face (no-change trials) or differ from it (change trials). Change and no-change trials were equally likely and randomly interleaved throughout the task. In the change trials, the new face images were randomly selected from faces with the same memorability level, minimizing change responses driven by unidentified perceptual differences between highly memorable and less memorable faces.

#### 3.1.3. Procedure

The laboratory settings were the same as those in the Pilot experiment. Participants completed an experimental session of two tasks: a face VSTM change detection task and a face image familiarity rating task, with the order counterbalanced across participants.

##### VSTM change detection task.

**Fig. 2.**
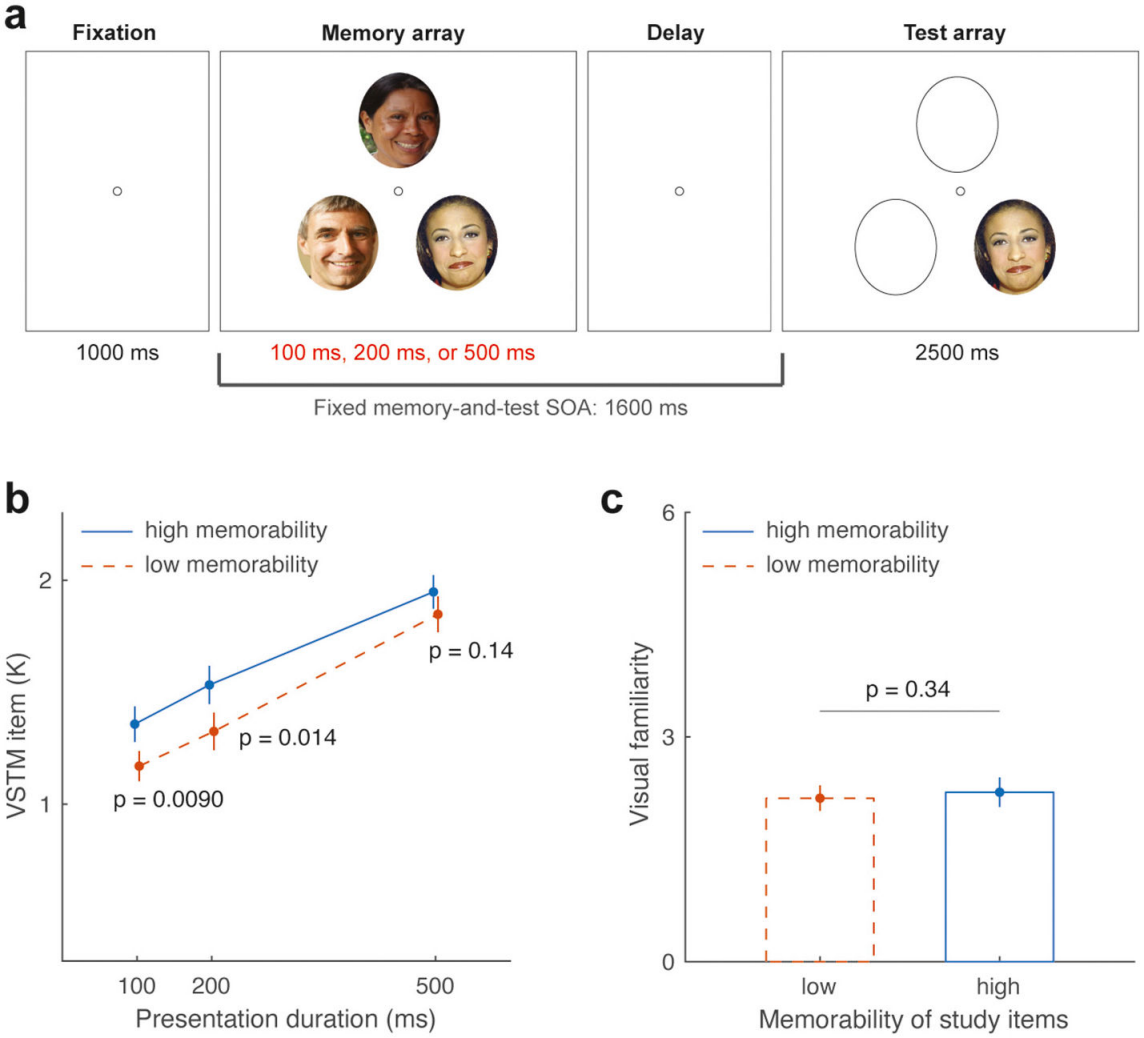
Early influence of visual memorability on VSTM formation in Experiment 1. (a) An example trial of Experiment 1 involves the brief presentation of three faces (either all low or all high memorability) for different durations (100, 200, or 500 ms), followed by a delay period with a fixed memory-and-test SOA of 1600 ms. Participants tried to make a single-probe change detection judgment within a response time window of 2500 ms during the presentation of the test array. In each trial, the face images presenting in the memory array would not be repeated. The display screen’s background color during the experiment was black, although this figure shows a white background for printing efficiency (the same for subsequent figures). (b) Across varying durations of memory presentation, participants show significantly enhanced memory for high-memorability faces as compared with low-memorability faces. This effect is more prominent at shorter presentation durations (e.g., 100/200 ms). (c) The early advantage of memorability cannot be attributed to the participants’ visual familiarity with these images, as no statistically significant difference is detected in familiarity ratings between the high- and low-memorability conditions. The error bars represent the standard error of the mean. All face images used in the current figure and in the rest of the figures are under a Creative Commons license for noncommercial use. Details about the copyright information of these face images can be found in the Supplementary Materials.

Each trial began with a 1000 ms presentation of a central fixation circle, followed by the memory array comprising faces displayed for one of three different durations: 100 ms, 200 ms, or 500 ms (see Fig. 2a). Participants were instructed to memorize a face during a fixed memory-and-test SOA of 1600 ms. Subsequently, within a response window of 2500 ms, participants provided a change detection response based on the face image presented in the test display. They indicated whether the tested face was “same” by pressing the “F” key or “different” by pressing the “J” key on the keyboard. Accuracy was emphasized over response speed. During the initial practice phase of 10–20 trials, participants received performance feedback, whereas no feedback was provided during the rest of the experiment. The experiment encompassed a total of 360 trials, divided into 6 blocks of 60 trials. Within each block, all experimental factors, including the two levels of face memorability and the three memory presentation durations, were randomly intermixed. A brief break was given between each block.

##### Face familiarity rating task.

To evaluate the participants’ visual familiarity with the experimental stimuli, a face image familiarity rating task was administered using a subset of face images (Xie & Zhang, 2017b). For each participant, a set of 20 high-memorability and 20 low-memorability face images were randomly selected from the pre-determined face pool (58 high-memorability faces and 58 low-memorability faces). For each trial, participants were presented with one of the randomly chosen faces and were asked to rate its visual familiarity based on their past experience prior to the study. Participants provided an untimed response using a Likert scale ranging from 1 (“unfamiliar”) to 6 (“familiar”), displayed at the bottom of the presented face image.

#### 3.1.4. Data analysis

Participants’ performance on the change detection task was assessed using Cowan’s K, which was calculated as the set size (3) multiplied by the difference between the hit rate and the false alarm rate (Rouder, Morey, Morey, & Cowan, 2011). This measure is closely associated with the number of items successfully retained in VSTM (Cowan, 2001). As this measure accounts for false recognition, it captures participants’ overall VSTM task performance after correcting for potential response biases. This measure was analyzed using a repeated-measures ANOVA with a 2 (memorability: high vs. low) × 3 (memory array presentation duration: 100 ms vs. 200 ms vs. 500 ms) design. Planned contrasts were performed using paired-samples *t*-tests to compare the high- and low-memorability conditions (Furr & Rosenthal, 2003; Rosenthal, Rosnow, & Rubin, 2000). All *p* values reported here and for the rest of the study are two-tailed. We also compared the participants’ familiarity ratings for high- and low -memorability face images to confirm that the observed effects were specific to visual memorability rather than stimulus familiarity.

### 3.2. Results

As the presentation duration increased, the number of VSTM items participants remembered also increased (see Fig. 2b), replicating some classic findings (e.g., for within category items in Quirk, Adam, & Vogel, 2020).This was supported by a significant main effect of the presentation duration (mean Cowan’s Ks for the 100 ms, 200 ms, and 500 ms conditions: 1.27 ± 0.07, 1.43 ± 0.07, 1.90 ± 0.07, respectively; F(2,78) = 66.91, *p* < 0.001, $\eta_{p}^{2}=0.63$). Of primary interest, we found that participants in general showed better change detection task performance for memorable items relative to forgettable ones, which was supported by a significant main effect of the memorability condition on Cowan’s K (mean Cowan’s Ks for high- vs. low-memorability conditions: 1.61 ± 0.06 vs. 1.44 ± 0.07; F (1,39) = 17.78, p < 0.001, $\eta_{p}^{2}=0.31$).

Follow-up planned contrast between memorability conditions at each stimulus presentation duration suggested the emergence of the memorability benefit as early as in the 100 ms presentation duration condition (mean Cowan’s Ks for high- vs. low-memorability images: 1.36 ± 0.08 vs. 1.17 ± 0.07, t(39) = 2.75,*p* = 0.009, Cohen’s d = 0.44). This memorability benefit extends to the 200 ms presentation duration condition (mean Cowan’s Ks for high- vs. low-memorability images: 1.53 ± 0.09 vs. 1.33 ± 0.08, t(39) = 2.58, *p* = 0.014, Cohen’s d = 0.41), but it diminishes at the 500 ms presentation duration condition (mean Cowan’s Ks for high- vs. low-memorability images: 1.95 ± 0.075 vs. 1.85 ± 0.08; e.g., 500 ms: t(39) = 1.50, *p* = 0.14, Cohen’s d = 0.24). However, the interaction effect between stimulus memorability and presentation duration was not statistically significant (F (2,78) = 0.60, *p* = 0.55, $\eta_{p}^{2}=0.015$).

The observed memorability benefits also could not be accounted for by the participants’ familiarity with task stimuli, as both high- and low-memorability faces have a similar level of familiarity rating, on average, based on a 6-point scale (high- vs. low-memorability images: 2.26 ± 0.20 vs. 2.18 ± 0.17, t(39) = 0.97, *p* = 0.34, Cohen’s d = 0.15; see Fig. 2c). Furthermore, as participants have not been exposed to these face images before the current experiments, these early memorability benefits could not be accounted for by their familiarity with the presented faces.

### 3.3. Discussion

The results in Experiment 1 show that memorability benefits on VSTM formation emerge within a timeframe of approximately 100 ms to 200 ms following stimulus onset. These findings indicate that the advantages of memorability become apparent even prior to the allocation of late-stage VSTM resources, which typically occurs at around 200 ms to 500 ms (Long et al., 2020; Ye et al., 2017, 2019). These rapid and early benefits at the perceptual encoding stage are less susceptible to contamination by post-perceptual influences that may arise with longer stimulus presentation durations (van Zoest, Hunt, & Kingstone, 2010). During longer presentation durations, participants may have more time to encode/process additional features of a low-memorability face, thereby potentially reducing the effect size between the high- and low-memorability conditions. Conversely, shorter presentation durations impose a higher demand for encoding efficiency (Hao et al., 2018). Therefore, while the memorability benefit may not vanish under conditions of longer presentation durations, it may become less robust due to the introduction of additional factors as more perceptual encoding time is permitted. These findings align with the interpretation that visual memorability may reflect the prioritization of specific perceptual features in the visual system to support memory formation (Rust & Mehrpour, 2020), especially when early perceptual processing is constrained by stimulus presentation duration. However, using the presentation duration as an experimental approach to investigate the VSTM formation process has known limitations.

One limitation is that the formation of VSTM may commence as soon as the stimulus presentation begins and may not be immediately disrupted after the stimulus offset (Enns & Di Lollo, 2000). Previous studies have shown that, in the absence of a post-stimulus mask, stimulus information can still be available via visual persistence for ~100 ms to 200 ms after the stimulus offset (Averbach & Coriell, 1961; Brockmole, Wang, & Irwin, 2002; Coltheart, 1980; Di Lollo & Dixon, 1988). Consequently, visual memorability could solely influence the initial representation boost at the perceptual encoding stage, or it could also impact the early stages of VSTM consolidation. This uncertainty can be addressed using alternative approaches to explore the process of VSTM formation.

For instance, previous studies have disrupted the process of VSTM consolidation by presenting random pattern masks at various memory-and-mask SOAs following a fixed presentation duration (Ricker & Sandry, 2018; Vogel et al., 2006; Xie & Zhang, 2017c; Zhang & Luck, 2008). This approach has enabled researchers to distinguish between time-dependent modulations attributed to changes in VSTM consolidation speed driven by visual familiarity or a perceptual encoding benefit that results in a constant probabilistic increase in memory encoding likelihood (Xie et al., 2022; Xie & Zhang, 2017c). By leveraging this paradigm, our aim in the subsequent experiment is to determine whether visual memorability elicits a constant increase or a time-dependent change in task performance across different time points during the VSTM retention interval.

## 4. Experiment 2

To investigate how visual memorability influences VSTM consolidation, Experiment 2 used randomly selected faces as masking stimuli inserted between the memory and test arrays at various memory-and-mask SOAs to disrupt the VSTM consolidation process. Building on the findings from Experiment 1, we used a fixed presentation duration of 150 ms (i.e., the average of 100 and 200 ms) to control for the effect of presentation duration on task performance. We expected that participants would still show superior change detection for high-memorability faces than for low-memorability faces at this presentation duration. We also introduced the masking stimuli either immediately or after a short delay to interrupt different stages of VSTM consolidation. Memory-and-mask SOAs of 383 ms and 617 ms were selected to correspond to the mid to later stages of VSTM consolidation based on previous estimates derived from studies employing diverse visual stimuli, such as simple features (Vogel et al., 2006) and complex visual items (Ngiam et al., 2019; Xie & Zhang, 2017c). Our experimental design did not incorporate a no-mask condition for comparison in Experiment 2. This setup was based on results from Experiment 1, from which we inferred that a significant memorability benefit would still be observed under a no-mask condition when stimuli were presented for a fixed duration of 150 ms.

**Fig. 3.**
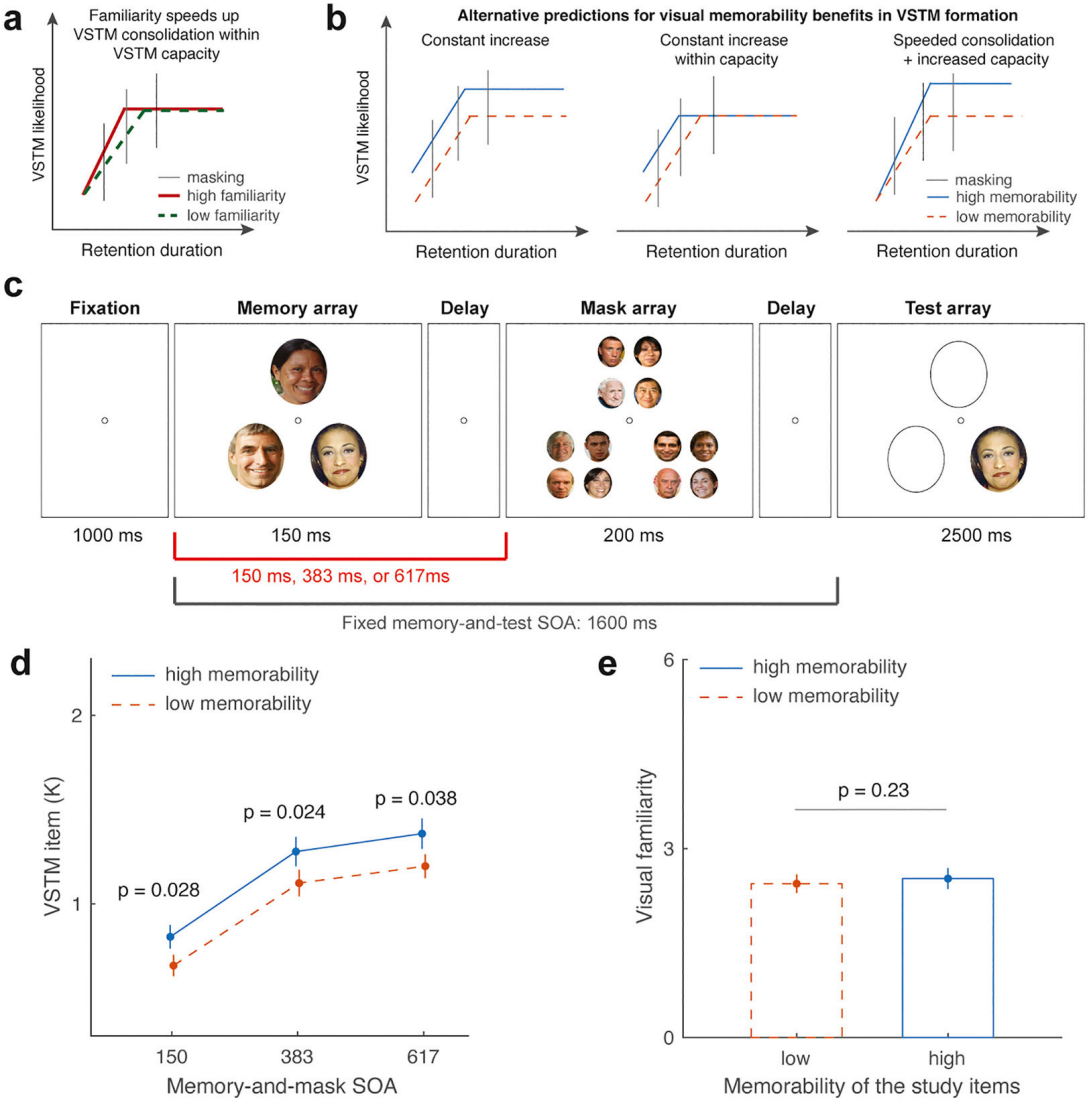
Constant boost of the early influence of visual memorability on VSTM formation in Experiment 2. (a) Previous research has demonstrated a time-dependent effect of participants’ familiarity with task stimuli on VSTM formation, where increasingly more familiar items can be encoded into VSTM over time within a storage capacity limit (Xie & Zhang, 2022). These effects can be tested by masking the VSTM consolidation process at different memory-and-mask stimulus onset asynchronies (SOAs) (see *light black* lines). (b) Based on the same approach, the impact of visual memorability on VSTM formation may differ from these well-documented familiarity effects. For example, visual memorability may lead to an early, constant perceptual encoding benefit that extends above the initial storage capacity (*left* panel) or operates within it (*middle* panel). Additionally, visual memorability may also exhibit an additive effect on both VSTM consolidation speed and storage capacity (*right* panel). (c) An example trial of Experiment 2, where participants try to remember either low or high-memorability faces for a fixed duration, followed by random-face masks at different memory-and-mask SOAs (150, 383, or 617 ms). (d) Across memory-and-mask SOAs, participants show significantly better memory for high-memorability faces than for low-memorability faces. As the presentation duration is fixed, the memorability effects are similar in size across memory-and-mask SOA conditions. (e) Again, these effects cannot be attributed to differences in participants’ visual familiarity for these face images. Error bars represent the standard error of the mean.

If visual memorability amplifies representation at the perceptual encoding stage during VSTM formation, we expected to see a higher likelihood of successful VSTM formation for images with higher (vs. lower) memorability across different memory-and-mask SOAs. This prediction is grounded in an early model on visual processing (Bundesen, 1990), where an advantage in perceptual processing should reduce the time required to form a robust percept that is resistant to subsequent masking. Consequently, this earlier perceptual encoding effect should remain unaffected by the timing of post-stimulus masking, as indicated by an upward shift in the VSTM consolidation curve (as illustrated in the *left* and *middle* panels of Fig. 3b). Second, this perceptual encoding benefit may plausibly be constrained by a rigid storage capacity (*middle* panel in Fig. 3b). However, we find this scenario less likely, based on the results of Experiment 1 and previous research (Gillies et al., 2023), in which participants generally demonstrated superior memory performance for high-memorability items in VSTM than for low-memorability items. Therefore, the possibility remains that visual memorability accelerates VSTM consolidation while simultaneously enhancing storage capacity, thereby resulting in an increasing number of items remembered in VSTM as the memory-and-mask SOA increases (as depicted in the *right* panel of Fig. 3b). As a preview, our data do not support this third prediction; rather, they suggest a consistent representation boost from the early perceptual encoding stage across different memory-and-mask SOAs (as illustrated in the *left* panel of Fig. 3b). Again, as in Experiment 1, we asked participants to provide their ratings of familiarity with the task stimuli to rule out the possibility that any observed effects were driven by their familiarity with the task stimuli.

### 4.1. Methods

#### 4.1.1. Participants

Another group of 40 Chinese college students (6 males, 34 females, 0 others; mean age = 20.45 ± 0.39 years) participated in this experiment with monetary compensation at Sichuan Normal University. All participants reported having normal or corrected-to-normal vision and provided written informed consent. None of these participants had previously participated in any of the previous experiments.

#### 4.1.2. Materials

The same high- and low-memorability faces in Experiment 1 were used for the memory and test arrays in this experiment. To mask each memory item, we selected 4 additional medium-memorability faces obtained from our Pilot experiment to ensure no repetition with the faces used in the memory and test arrays (see Fig. 3c). In total, 80 medium-memorability faces were used to create 20 unique masking patterns. Consequently, the mask array consisted of 3 randomly selected masking patterns positioned at the original locations of the memory items. Each masking pattern (measuring 2.6° × 3.0° of visual angle) comprised 4 smaller face images (measuring 1.3° × 1.5° of visual angle).

#### 4.1.3. Procedure

Participants performed the same experimental procedure as in Experiment 1, again consisting of a VSTM change detection task and a face familiarity rating task, but with the following modifications. In the current change detection task (see Fig. 3c), participants were first presented with a 1000 ms fixation interval, followed by the display of 3 faces on the screen for 150 ms. Subsequently, a mask display consisting of facial images positioned at the same locations as the faces in the memory array was presented for 200 ms, with an SOA between the memory array and the mask display of either 150 ms, 383 ms, or 617 ms. After the mask display, a test display was shown with a fixed SOA between the memory and test displays of 1600 ms. Participants were instructed to indicate whether the test display showed the same or different faces compared to the memory array within a response time window of 2500 ms, similar to Experiment 1. Accuracy was emphasized over speed in their responses. Each participant completed a total of 360 trials, divided into 6 blocks of 60 trials. All experimental factors (memorability: high vs. low; memory-and-mask SOA: 150 ms, 383 ms, vs. 617 ms) were randomly mixed within each block. Short breaks were provided between blocks to ensure the participants’ comfort and engagement. Additionally, in the modified face familiarity rating task, each participant rated the familiarity of all 58 high-memorability images and all 58 low-memorability images, resulting in a total of 116 trials.

#### 4.1.4. Data analysis

A repeated-measures analysis of variance (ANOVA) with a 2 (memorability: high vs. low) × 3 (memory-and-mask SOA: 150 ms vs. 383 ms vs. 617 ms) design was performed to examine the variations in Cowan’s K across different experimental conditions. We also computed the difference in K values between the 383 ms and 150 ms SOA conditions to serve as an estimate of the speed of early-stage VSTM consolidation. Similarly, the difference in K values between the 617 ms and 383 ms SOA conditions was calculated as an indicator of the speed of late-stage VSTM consolidation.

### 4.2. Results

As memory-and-mask SOA increased, the number of retained VSTM items estimated from the change detection task also increased (Fig. 3d). This result was supported by a significant main effect of memory-and mask SOA (mean Cowan’s Ks for 150 ms, 383 ms, and 617 ms SOA conditions: 0.75 ± 0.05, 1.19 ± 0.07, and 1.29 ± 0.06, respectively; F (2,78) =72.37, *p* < 0.01, $\eta_{p}^{2}=0.65$). While the number of retained VSTM items increased significantly from the 150 ms to 383 ms memory-and-mask SOA conditions (t(39) = 8.46, *p* < 0.001, Cohen’s d = 1.34), the increase in remembered VSTM items from 383 ms to 617 ms condition memory-and-mask SOA conditions was much attenuated (t(39) = 2.10, *p* = 0.042, Cohen’s d = 0.33). These results suggested the period from 150 ms to 383 ms remains a critical VSTM formation period, whereas the VSTM formation may reach a later stage after 383 ms. These results replicated previous findings using this paradigm (Ngiam et al., 2019; Vogel et al., 2006; Xie et al., 2022; Xie & Zhang, 2017c), suggesting the effectiveness of the current masking manipulation.

Of primary interest, we found a significant main effect of memorability on Cowan’s K, in that memorable items had a higher likelihood of being encoded into VSTM across memory-and-mask SOAs (mean Cowan’s Ks for high- vs. low-memorability conditions: 1.16 ± 0.06 vs. 0.99 ± 0.05; F (1,39) = 12.44, *p* = 0.001, $\eta_{p}^{2}=0.24$). This observation is in line with the lack of a significant interaction effect between stimulus memorability and memory-and-mask SOA (F (2,78) = 0.02, *p* = 0.98, $\eta_{p}^{2}<0.001$). A closer inspection, based on the effect sizes of the memorability benefit on the number of remembered VSTM items across memory-and-mask SOA conditions, also revealed similar observations. In particular, the effect size of memorability benefit was numerically similar in the 150 ms condition (t(39) = 2.28, *p* = 0.028, Cohen’s d = 0.36), in the 383 ms condition (t(39) = 2.36, *p* = 0.024, Cohen’s d = 0.37), and in the 617 ms condition (t(39) = 2.15, *p* = 0.038, Cohen’s d = 0.34).

Again, these results could not be accounted for by variability in participants’ familiarity with the face images, as the participants’ familiarity rating scores were again statistically comparable between the high- and low-memorability faces (high- vs. low-memorability: 2.52 ± 0.17 vs. 2.44 ± 0.16: t(39) = 1.21, *p* = 0.23, Cohen’s d = 0.19; Fig. 3e). Importantly, when we combined the familiarity rating data from both Experiments 1 and 2 to improve statistical power (*n* = 80), there remained no statistically significant difference in participants’ perceived familiarity between high- and low-memorability faces (high- vs. low-memorability: 2.39 ± 0.13 vs. 2.31 ± 0.12: t(79) = 1.52, *p* = 0.13, Cohen’s d = 0.17). These results again highlight that observer-level familiarity with the task stimuli is not a primary factor driving participants’ advantageous VSTM task performance for high-memorability stimuli in the current study.

### 4.3. Discussion

In Experiment 2, we once again found that visual memorability exerts an early influence on VSTM formation within as early as 150 ms of stimulus presentation, despite the presence of post-stimulus masking. Furthermore, the effect of memorability on VSTM formation appears to be consistent across different durations of the memory-and-mask SOA, indicating that the size of this effect remains relatively stable during the VSTM retention interval. These results contrast with previous studies that have emphasized the time-dependent influence of stimulus familiarity on VSTM formation within the VSTM capacity limit (Ngiam et al., 2019; Xie & Zhang, 2017c). Therefore, the present findings support the notion that the benefit of memorability to VSTM formation is aligned more closely with a perceptual encoding benefit than with a process of speeded VSTM consolidation.

Our findings underscore the different implications of a stimulus’s visual memorability and an observer’s familiarity with the stimulus in visual memory formation. Behaviorally, both visual memorability and participants’ familiarity with face images have been shown to influence performance in VSTM (Buttle & Raymond, 2003; Gillies et al., 2023), making it challenging to disentangle their unique contributions. By controlling for visual familiarity through the selection of unfamiliar faces for our research population and confirming this through participants’ ratings, our data obtained from both Experiments 1 and 2 suggest that visual memorability, as a stimulus-driven phenomenon, can influence VSTM even in scenarios when the observer-level familiarity effect is more strictly controlled. Furthermore, our results suggest that memorability and familiarity can have distinct effects on VSTM formation. While high memorability could lead to a constant representation boost from the early perceptual encoding stage during VSTM formation as seen in Experiment 2, participants’ familiarity with the task stimuli could lead to speeded VSTM consolidation within a limited storage capacity (Xie & Zhang, 2022).

## 5. Experiment 3

The significant enhancement in VSTM task performance driven by visual memorability prompts us to investigate the durability of this effect within the current experimental framework. It remains unclear whether the early perceptual benefits driven by visual memorability in VSTM observed in Experiments 1 and 2 can be sustained over time and even account for the subsequent formation of robust VLTM representations. Conceptually, because VSTM plays a critical role in the establishment of VLTM (Atkinson & Shiffrin, 1968; Fukuda & Vogel, 2019), the potent VSTM representations formed at the early perceptual encoding stage may naturally facilitate the subsequent formation of VLTM. However, this memorability benefit across VSTM and VLTM is not a given, considering these following alternatives.

First, VSTM is known to exhibit rapid decay within a few seconds, regardless of whether this decay manifests as a sudden or gradual loss of information (Schneegans & Bays, 2018; Zhang & Luck, 2009). It remains unclear whether the enhancement of VSTM due to visual memorability is transient or would persist over time. To address this, in Experiment 3, participants were instructed to remember an identical set of three face images, each varying in terms of high or low memorability. The retention interval was manipulated to be 1400 ms, 4000 ms, or 10,000 ms. Our goal was to examine whether the initial perceptual enhancement of VSTM driven by visual memorability could endure a prolonged retention interval, particularly up to 10,000 ms. If the effect of memorability is short-lived, the advantage conferred by visual memorability in VSTM decay should diminish over time, resulting in a baseline level of remembered VSTM items at the extended delay. Alternatively, if visual memorability leads to a perceptual encoding benefit in VSTM formation, these enhanced VSTM representations might be shielded from decay or interference during the retention interval. Consequently, a memorability benefit should still be observed, even at the extended delay.

Second, although previous research has highlighted the stability of memorability estimate in VLTM across long intervals (Goetschalckx et al., 2018), the previous evidence is mixed regarding the relationship between the memorability benefit in VSTM and that in VLTM. For example, using face images as task stimuli, past research has shown that participants’ VSTM predicts their VLTM task performance only when faces with a certain memorability level are used (Gillies et al., 2023). Furthermore, VSTM is well established to act as a gatekeeper for VLTM formation (Fukuda & Vogel, 2019); therefore, these previous findings could reflect either an intrinsic relationship between the individual differences in VSTM and VLTM task performance or a genuine association between the additional benefit of memorability in VSTM formation and a corresponding benefit in VLTM. To clarify this, we administered a surprise visual recognition test to participants on the second day of Experiment 3 using stimuli previously presented in the VSTM task. To factor out individual differences and capture within-subject effects, we assessed whether or not the memorability boost in VSTM carries over to VLTM formation for each participant through an analysis of repeated-measured correlation between participants’ VSTM and VLTM task performance for task stimuli across different memorability levels (Bakdash & Marusich, 2017; Xie & Zhang, 2023a).

### 5.1. Methods

#### 5.1.1. Participants

A different group of 40 Chinese college students (7 males, 33 females, 0 others; mean age 19.85 ± 0.29 years) participated in the two-session experiment with monetary compensation at Sichuan Normal University. The 2 sessions of the experiment were separated by approximately 24 h (e.g., testing at 10 a.m. on Day 1 would be followed by a session scheduled for 10 a.m. on Day 2). Prior to the study, all participants confirmed having normal or corrected-to-normal vision and provided written informed consent.

#### 5.1.2. Materials

The task materials were identical to those of the previous experiments.

#### 5.1.3. Procedure

Each participant took part in a two-session study, comprising a VSTM task on Day 1 and a surprise VLTM recognition task, consisting solely of a test phase, on Day 2. While participants were informed that the experiment spanned two consecutive days, they were not provided exact details regarding the content or nature of the Day 2 experiment while performing the tasks on Day 1.

##### VSTM change detection task.

**Fig. 4.**
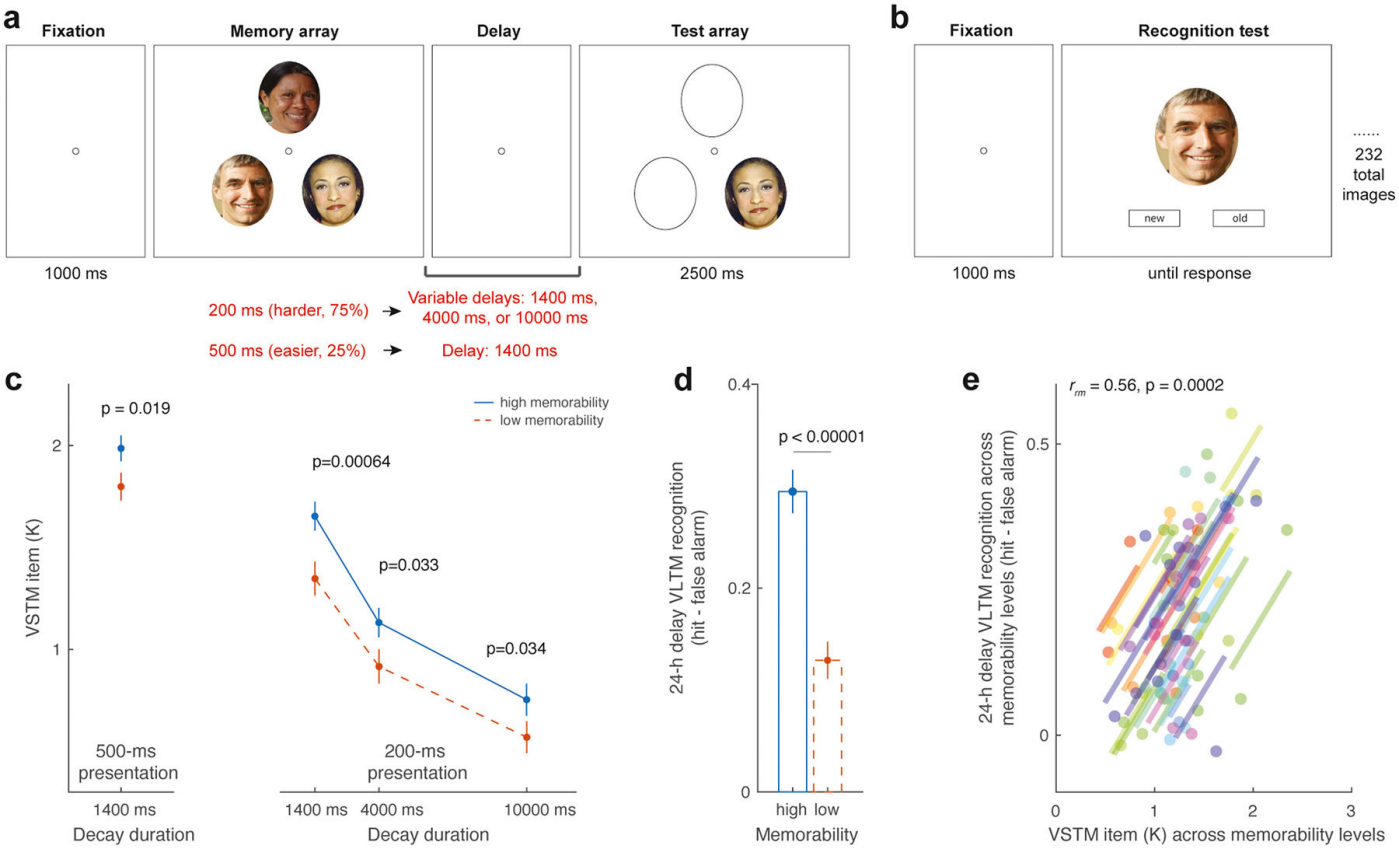
Long-lasting effects of visual memorability on visual memory formation for up to 24 h in Experiment 3. (a) An example trial of the VSTM task of Experiment 3, where the retention interval is manipulated to be 1400, 4000, or 10,000 ms with a 200 ms stimulus presentation duration for the difficult trials (75% of total). For the remaining easy trials (25% of total), the retention interval was fixed at 1400 ms with a 500 ms stimulus presentation duration. The inclusion of both easy and difficult trials helps to encourage the participants to complete the study despite the increased task difficulty. (b) An example trial of the VLTM recognition task after a ~ 24 h delay. (c) Participants reliably remember more high-memorability faces than low-memorability faces in the VSTM task across various task difficulty levels and VSTM retention intervals. Of primary interest, the memorability effect appears to be stronger when VSTM items are presented with a short presentation duration, even when the delay interval is fixed at 1400 ms (i.e., 200 ms vs. 500 ms stimulus presentation conditions). (d) This memorability benefit persists following a ~ 24-h delay, manifested as a higher corrected accuracy (i.e., hit – false alarm) in VLTM recognition task performance for high- relative to low-memorability face images. (e) Critically, repeated-measures correlation analysis reveals that the memorability benefit observed in the VSTM task can directly predict the memorability benefit observed in the VLTM task among participants. Individual data across memorability levels (*dots*) from each participant are color-coded. The solid lines represent linear fits of the data, capturing the maximum amount of variance between VSTM and VLTM task performance within participants. Error bars represent the standard error of the mean.

The task used in this experiment closely resembled that of Experiment 1, with the following modifications (Fig. 4a). First, the study items were presented for a fixed duration of 200 ms. Second, the delay interval was manipulated as 1400 ms, 4000 ms, or 10,000 ms, similar to the interval manipulation in the previous research (Zhang & Luck, 2009). No masking stimuli were administered during the retention interval. Third, to account for the increased task difficulty associated with the inclusion of delay manipulation, occasional easy trials were introduced, involving a presentation duration of 500 ms and a memory-and-test duration of 1400 ms to encourage participants’ completion of the experiment. Each participant completed 384 trials in the primary task, based on a 2 (memorability: high vs. low) × 4 (condition: short delay, moderate delay, long delay, and easy) within-subject design. These trials were distributed across 8 blocks of 48 trials, with the experimental factors randomly intermixed. Short breaks were provided between the blocks.

##### VLTM face recognition task.

On the second day, participants were asked to conduct a surprise recognition test to assess their memory for the faces they encountered on the first day. Each trial commenced with the presentation of a central fixation point on the screen for 1000 ms, followed by the display of a single face image at the center of the screen (Fig. 4b). Participants were instructed to determine whether the presented face image had been previously shown on Day 1 (“old,” responding with the “F” key), or if it had not been presented during the course of the experiment (“new,” responding with the “J” key). Emphasis was placed on accuracy rather than speed; thus, the stimuli remained visible on the screen until a response was made by the participant. In half of the trials, the face image corresponded to one of the 116 images shown during the VSTM task. In the remaining half of the trials, the face image was a new image randomly selected from the medium-memorability facial image database, as determined during our Pilot experiment. Overall, the participants completed a total of 232 trials, with 58 trials involving old high-memorability faces, 58 trials involving old low-memorability faces, and the remaining 116 trials featuring new faces that were not shown previously. The presentation order of these trial types was randomized.

#### 5.1.4. Data analysis

The data analysis procedure for the VSTM experiment closely followed that of the previous experiments. Given our focus on examining how memorability impacts VSTM decay, our primary analysis only considered the trials involving the fixed presentation duration of 200 ms with variable delays. Therefore, the participants’ performance in the easier condition (500 ms presentation duration) was analyzed separately. For the additional VLTM recognition task, participants’ performance was evaluated separately for the high-memorability and low memorability conditions using “hit – false alarm” as the measure. Here, the hit rate was calculated based on the accurate detection of previously presented faces from the previous day, while the false alarm rate was determined by the average tendency to mistakenly identify any of the 116 new faces as “old.” By incorporating this calculation, we accounted for potential response biases in recognition accuracy. These bias-corrected recognition memory measures were then compared across the memorability conditions to assess the impact of memorability on VLTM recognition. We also examined how the memorability benefit in VSTM can be translated into subsequent VLTM recognition within participants through a repeated-measures correlation, namely r_rm_, to capture shared variances in participants’ task performance across VSTM and VLTM. This approach factors out individual differences in overall task performance and mitigates concerns associated with the use of difference scores when examining the relationship between two experimental effects (Bakdash & Marusich, 2017; Xie & Zhang, 2023a).

### 5.2. Results

As a vigilance test, the participants’ performance on easy trials (i.e., 500 ms presentation duration with 1400 ms delay period) matched with findings from Experiment 1, such that the number of remembered VSTM items was close to 2 items on average across memorability conditions (1.89 ± 0.05). Furthermore, we found that participants remembered significantly more high-memorability faces than low-memorability ones (high vs. low memorability: 1.98 ± 0.06 vs. 1.79 ± 0.06, t(39) = 2.45, *p* = 0.019, Cohen’s d = 0.39; Fig. 4c). This finding is not at odds with the results from Experiment 1, considering that memorability benefits emerging early with shorter presentation durations may also manifest under conditions with longer presentation times (Ye et al., 2017). Supporting this interpretation, within the current experiment, participants’ VSTM task performance was better for high-memorability faces on harder trials with a 200 ms presentation duration and the same delay period of 1400 ms (high vs. low memorability: 1.65 ± 0.07 vs. 1.34 ± 0.08, t(39) = 3.71, *p* < 0.001, Cohen’s d = 0.59).

Of primary interest, when the presentation duration was fixed at 200 ms, we found that increases in the VSTM delay duration were associated with decreases in the number of retained VSTM items across both highl- and low-memorability conditions (Fig. 4d). This was supported by a significant main effect of retention intervals (1400 ms, 4000 ms, 10,000 ms: 1.50 ± 0.07, 1.02 ± 0.06, and 0.66 ± 0.07, respectively; F (2,78) = 86.64, p < 0.001, $\eta_{p}^{2}=0.69$). Furthermore, we found a significant main effect of memorability on Cowan’s K, as memorable items had a higher likelihood of being encoded into VSTM across delay duration (high- vs. low-memorability images: 1.17 ± 0.06 vs. 0.94 ± 0.06; F (1,39) = 15.92, p < 0.001, $\eta_{p}^{2}=0.29$). No significant interaction was noted between stimulus memorability and the VSTM retention interval (F(2,78) = 0.62, *p* = 0.54, $\eta_{p}^{2}=0.016$), suggesting that the memorability benefit on Cowan’s K was similar across retention interval conditions (1400 ms delay: t(39) = 3.71, p < 0.001, Cohen’s d = 0.59; 4000 ms delay: t(39) = 2.21, *p* = 0.033, Cohen’s d = 0.35; 10,000 ms delay: t(39) = 2.20, *p* = 0.034, Cohen’s d = 0.35).

We also investigated participants’ VLTM recognition task performance after the ~24 h delay. Each face, regardless of its memorability level, had an equal likelihood of being presented and tested across VSTM conditions on Day 1; therefore, if no additional factors other than visual memorability were affecting memory likelihood, we expected that the likelihood of remembering a given face image should be the same across all faces. In contrast to this prediction, the participants showed a higher corrected hit rate (“hit – false alarm”) for high-memorability faces than for low-memorability faces (high vs. low memorability: 0.30 ± 0.02 vs.0.13 ± 0.02; t (39) =10.65, p < 0.001, Cohen’s d = 1.68). These results were in line with the findings from our Pilot experiment and from the previous research, suggesting the robustness of these memorability effects in the VLTM domain.

Finally, we then investigated the relationship between the memorability benefit for VSTM (Cowan’s Ks averaged across all VSTM delay conditions) and that for VLTM (corrected hit rate, “hit – false alarm”). We found a significant correlation between the number of items the participants remembered in VSTM and VLTM recognition performance across memorability conditions within participants (*r*_*rm*_ = 0.56, *p* < 0.001; see Fig. 4e). As this repeated-measures correlation metric factors out individual differences in average task performance across experimental conditions, it highlighted that the memorability benefit in VSTM could be directly used to predict the magnitude of the subsequent memorability benefit in VLTM among participants.

### 5.3. Discussion

Our data in Experiment 3 provide compelling evidence for the enduring impact of visual memorability on visual memory formation. Visual memorability has early influences on VSTM while also contributing to the development of more resilient VSTM representations during extended retention intervals. As a result, the advantage of memorability in VSTM is effectively transferred to VLTM, leading to improved performance in visual recognition tasks overnight. These findings suggest that the impact of visual memorability on VLTM recognition may manifest early on from the initial stages of perceptual encoding and persist throughout the consolidation process.

Our findings raise intriguing questions about the origin of these enduring memorability benefits on visual memory. For example, previous research has suggested that memorable visual items may compete more effectively for cognitive resources to support successful VSTM encoding and retention, or they may demonstrate greater resiliency against time-related factors that lead to the forgetting of VSTM representations (Gillies et al., 2023). However, our current findings, in conjunction with the results from Experiments 1 and 2, do not fully align with these explanations. Instead, a more parsimonious account emerges, indicating that visual memorability may lead to early perceptual encoding benefits that can carry over into later cognitive processes that encompass both VSTM and VLTM. This interpretation rests upon the intuition that remembered VSTM items may exhibit a similar forgetting rate regardless of their original memorability levels; consequently, differences in task performance may be attributed to early processes related to the initial encoding rather than to subsequent control-related or forgetting-related mechanisms (Bainbridge, 2020). Moreover, given that participants were asked to remember study items with the same level of memorability (thereby experiencing less direct within-trial competition among items) and that memorability benefits did not amplify as the VSTM retention interval increased, the alternative possibility regarding attention selection during VSTM encoding could not account for the current findings.

It is noteworthy that we observed a memorability benefit across VSTM and VLTM in Experiment 3 by categorizing memory images into two types: those with high memorability and those with low memorability. Each face image in the VSTM change detection task had an equal chance of being drawn from either the high or low memorability image pools. Consequently, any differences in subsequent memory performance between memorability conditions are unlikely to be driven by variations in other task parameters at the level of individual items (e.g., the number of times each item was presented in the VSTM task or whether faces were used as changed or unchanged items). Therefore, our aim was to demonstrate the relationship between VSTM and VLTM effects at the condition level within each participant – an approach that can mitigate analytical challenges in balancing various task-related parameters mentioned above. Future studies with designs optimized for item-level analysis may further reveal participants’ memory task performance with individual images across both VSTM and VLTM.

## 6. General discussion

The present study systematically demonstrates the early and lasting impacts of visual memorability on visual memory formation through four experiments. In the Pilot experiment, we first generalized past findings to a different research population and selected a set of face images with varying degrees of memorability that were presumably equally (un)familiar to our participants. Using this verified image set, we find that the benefit of visual memorability on VSTM emerges within 100–200 ms following stimulus onset (Experiment 1) and survives post-stimulus masking during VSTM retention (Experiment 2). This early memorability benefit is also resistant to short- and long-term forgetting over delays from a few seconds up to 24 h (Experiment 3). Of particular importance, the early memorability benefit observed in VSTM can directly predict the subsequent memorability benefit in VLTM within participants (Experiment 3). This finding emphasizes the early emergence of a substantial amount of predictable variance in memory task performance since initial perceptual encoding. Taken together, the findings converge on a parsimonious perceptual encoding benefit account for the visual memorability benefit in visual memory formation. As such, our results have significant implications regarding the role of visual memorability in understanding the intricate relationship between perception and memory in the visual domain.

Of primary interest, our data highlight that visual memorability, as a robust and generalizable visual memory phenomenon, has an early behavioral encoding benefit during visual memory tasks. Notably, while previous electrophysiology evidence has suggested that neural signals linked to memorability tend to manifest within the initial 100–200 ms following a stimulus onset and potentially precede signals associated with successful memory encoding (Mohsenzadeh et al., 2019), to our knowledge, no direct behavioral evidence has been provided to collaborate this conjecture. This gap in knowledge has persisted in part because previous research has primarily relied on memory tasks that do not typically impose a strong demand on perceptual encoding (e.g., a longer encoding duration of a few seconds with an unmasked inter-stimulus interval in an online setting). As a result, a question remains as to whether the profound and consistent behavioral effects associated with visual memorability observed in VLTM studies indeed emerge during early perceptual encoding or whether they can be attributed to some post-perceptual covariates, such as participants’ familiarity with the task stimuli and prolonged encoding time. By controlling for these post-perceptual encoding covariates, our data from Experiments 1 and 2 provide compelling evidence in support of the early perceptual encoding benefit of visual memorability. Our findings, therefore, replicate and extend the prior behavioral findings using the RSVP paradigm (Broers et al., 2018), by highlighting that the advantageous early visual processing for memorable images can directly account for the boosted visual memory formation across both VSTM and VLTM.

Furthermore, our findings in Experiment 2 present a stark contrast to the findings associated with observer-level familiarity effects using similar experimental approaches (Ngiam et al., 2019; Xie & Zhang, 2017c), highlighting a dissociation between a stimulus’s visual memorability and an observer’s familiarity with the stimulus in their respective impacts on visual memory formation. Behaviorally, both factors exhibit the potential to enhance performance in memory tasks, often creating challenges in disentangling them from one another. For instance, previous research has unveiled individuals’ superior memory for celebrity faces compared to unfamiliar faces (Jackson & Raymond, 2008), a phenomenon often attributed to a familiarity advantage. However, another plausible explanation is that certain aspects of these memory advantages might stem from the potentially heightened memorability exhibited by select individual celebrity faces. Therefore, the exploration of memorability may prompt a reevaluation of interpretations associated with certain previous research findings. In contrast to these prior studies, the control of participants’ familiarity with task content was achieved through a threefold approach in our current experiment, including (1) the use of face images from a dataset that is largely unseen by our research participants, (2) the exclusion of any celebrity faces, and (3) the confirmation based on participants’ subjective ratings. Consequently, the data derived from both Experiments 1 and 2 effectively elucidate that visual memorability, as an inherently stimulus-driven phenomenon, holds the capacity to influence VSTM, even in instances where observer-level familiarity effects have been more rigorously controlled.

More broadly speaking, our findings highlight a perceptual root of the memorability benefit to visual memory formation (Bainbridge, Dilks, & Oliva, 2017; Rust & Mehrpour, 2020). Moving beyond a modular perspective (Fodor, 1983), recent research emphasizes the overlap between perceptual and mnemonic functions at the computational and neural levels. For example, sensory processes involved in visual perception may directly support memory representation and retention (Scimeca, Kiyonaga, & D’Esposito, 2018; but see Xu, 2017). Conversely, long-term memory processes (e.g., semantic knowledge) can also penetrate into early visual perception (Xie & Zhang, 2023b). Importantly, the sharing mechanism between perception and memory driven by visual memorability is expected to be independent of contextual, attentional, or other control-related processes (Bainbridge, 2020). Although our current study has not manipulated these factors, they are expected to contribute equally to conditions involving high- and low-memorability images in our current randomized experiments. Therefore, our current findings should be orthogonal to these potential additional mechanisms (Gillies et al., 2023; Wakeland-Hart, Cao, DeBettencourt, Bainbridge, & Rosenberg, 2022). Furthermore, given the timescale of the current effects, we attribute the early representational boost conferred by visual memorability to a perceptual advantage, in line with prior interpretations (Rust & Mehrpour, 2020). Alternative interpretations, such as the rapid formation of concurrent VSTM and VLTM traces (Wickelgren, 1970; Wickelgren & Berian, 1971) or other “active–silent” mnemonic mechanisms (Stokes, 2015), might also contribute to the observed memorability benefits. However, these accounts impose additional assumptions that entail further justification. In light of our current findings, the perceptual encoding benefit appears to offer a more parsimonious and succinct explanation for the advantageous influence of visual memorability on visual memory formation.

In our study, we did not require participants to perform a concurrent articulatory suppression task during the VSTM task. Given that semantic analysis of faces occurs rapidly and often unconsciously (Axelrod, Bar, & Rees, 2014), another noteworthy aspect to consider is whether the effect of memorability on the VSTM performance we observed is attributed not to a perceptual encoding benefit but solely to the facilitation of early semantic processing. However, we find this explanation inadequate because of several reasons. First, a recent study by Lin et al. (2021) involved the removal of semantic information through phase scrambling, and it still observed a memorability effect for scrambled images. This suggests that the memorability effect can remain despite an interruption in an observer’s ability to generate a semantic label for scrambling images. Second, although human observers can rapidly employ semantic processing to categorize a face in terms of its gender or race, it takes much longer time and effort to encode a specific face (Cloutier, Mason, & Macrae, 2005; Hugenberg, Young, Bernstein, & Sacco, 2010), especially when there are three faces to remember simultaneously. As low- and high-memorability faces are matched in these major stimulus categories (e.g., gender or race; see Supplementary Table S1 for details), early semantic processing of major face categories, therefore, should not account for a significant amount of variance in the difference between low- and high-memorability conditions in the current study. Third, if the differences in VSTM performance for faces with varying memorability observed in our study were mainly due to the influence of more elaborated encoding (Craik & Lockhart, 1972), we would anticipate that participants with longer processing time should exhibit a greater memorability advantage. However, the diminishing memorability advantages as stimulus presentation time extended in Experiment 1 suggests against this alternative interpretation.

One important point to note is that human memories are often complex and can be retained in different formats (Liu et al., 2020; Xie & Zaghloul, 2021). Our current study does not diminish the significant impacts of an observer’s prior semantic knowledge or pre-existing VLTM on stimulus memorability and memory formation/retrieval processes. This understanding is particularly relevant when scaling up our knowledge of human cognition from well-controlled laboratory stimuli in one format to more naturalistic stimuli perceived and memorized with multiple formats (e.g., visual, auditory, tactile, etc.). Our current experimental approach is valuable for isolating individual determinants underlying the improved memory likelihood across VSTM and VLTM that are driven mostly by stimulus-related factors. This approach is complementary to others involving strict control of perceptual contributions to verbal memory retrieval (e.g., Xie, Bainbridge, et al., 2020).. Furthermore, by highlighting the early perceptual encoding benefit driven by stimulus-related factors, our findings suggest the possibility of modifying the basic properties of visual images to facilitate the formation of long-lasting visual memory (Khosla, Bainbridge, Torralba, & Oliva, 2013) —an applied domain that may have impacts on advertising and education (Borkin et al., 2013).

While we have generalized our major experimental results across various experimental conditions, indicating that memorability benefits in visual memory formation emerge at a short presentation duration (100–200 ms) with or without post-stimulus masking and with or without a prolonged delay, several constraints should be noted in the current study. First, although we used face images as the primary task stimuli, based on previous research (Bylinskii et al., 2015; Gillies et al., 2023; Rust & Mehrpour, 2020), we anticipate that our findings should be generalizable to other visual stimuli employing similar experimental setups. Future research may extend these findings to explore other visual contents (e.g., objects and scene images). Yet, the exact timing of the early perceptual boost of the memorability of these other visual contents on visual formation remains untested and therefore requires future investigation. Furthermore, as slight alterations in facial features (e.g., shape/size) can sometimes result in changes in memorability (Khosla et al., 2013), it remains an open question to what extent the memorability benefits of face images on VSTM and VLTM are dependent on viewpoint or perspective. Clarifying this issue is helpful for understanding the more lasting effects of face memorability in real-world settings, where faces are often viewed from different angles while preserving their high-level perceptual attributes^1^ across different contexts (e.g., Xie & Zhang, 2016). Finally, while our data suggest that memorable content may be processed in a more advantage state, it remains to be determined if these memorability benefits are linked with increased memory strength or fidelity across both VSTM and VLTM—a theoretically important issue concerning how memorable stimuli are represented in visual memory. Future research using signal detection models may be useful to address this question (Xie & Zhang, 2017a; Yonelinas, 2023).

In sum, the current study provides robust evidence for the early and lasting impacts of visual memorability on visual memory formation. These results cannot be explained by post-perceptual factors introduced by variations in participants’ familiarity with the task stimuli or a prolonged encoding time. Our data offer a parsimonious perceptual encoding benefit account for these memorability effects, adding to our understanding of the intricate relationship between perception and memory in the visual domain.

## Supplementary Material

SI

## Data Availability

The data are available through the Open Science Framework at https://osf.io/gcj5s/
