# Supplementary material for: Perceptual encoding benefit of visual memorability on visual memory formation
Source: Cognition. Author manuscript; Available in PMC 2024 Sep 3. (PMC11369960; doi:10.1016/j.cognition.2024.105810)
Supplement: SI [file NIHMS2016683-supplement-SI.pdf]

Supplementary materials

In **Pilot Experiment**, a total of 160 faces were selected from the 10K US Adult Faces Database (Bainbridge, et al., 2013), with each group consisting of 80 faces categorized as either high-memorability or low-memorability. The following comparison highlights various attributes of the two groups based on the database (**Table S1**). The high-memorability and low-memorability groups exhibit significant differences in memory Hit Rate (HR) and Memorable ratings, while endeavoring to maintain statistical matching across other key attributes as much as possible (e.g., False Alarm Rate, FAR, memorability).

Subsequently, we selected 58 high-memorability and 58 low-memorability images from the initial pool of 160 pictures based on the results of **Pilot Experiment**. The following comparison illustrates various attributes of the two groups based on the 10K US Adult Faces Database (**Table S2**). The two groups exhibit significant differences in memory Hit Rate and Memorable ratings, with key attributed statistically controlled (e.g., False Alarm Rate, FAR, memorability).

It is possible that certain individual attributes are not perfectly matched between the selected faces, such as the number of teeth showing and facial hairs, etc. Since memorability alone cannot fully account for these variables in the larger dataset, and these attributes may capture perceptual features inherent in the images, we have chosen not attempt to control for them. To ensure that participants in our current research population are unfamiliar with the chosen faces, we asked participants to provide ratings on their familiarity with these selected face images in both **Experiments 1** and **2**. The results indicate that across the 80 participants involved, our current sample is not very familiar with these chosen stimuli, regardless of their memorability levels (see **Results** in **Experiments 1** and **2** for details)

Table S1 Hit Rate (HR), False Alarm Rate (FAR)and demographic information for each group. Statistics present independent t-test or x^2 test values investigating the group differences.

Attributes		LOW (n = 80)	HIGH (n =80)	Statistics
Hit Rate (HR)	Mean	0.354225	0.66326	t (158) = -33.085, p <0.0001
	SD	0.062899	0.05499	
Memorable	Mean	2.7711	3.3084	t (158) = -11.580, p <0.0001
	SD	0.26197	0.32183	
False Alarm Rate (FAR)	Mean	0.12631	0.13516	t (158) =-0.941, p = 0.348
	SD	0.05965	0.05931	
Age	Mean	3.3398	3.2760	t(158) =0.469 ， p =0.640
	SD	0.82207	0.89596	
Gender	Female	36	36	
	Male	44	44	
Race	White	72	69	x^2 (3) = 0.607, p =0.895
	Black	4	6	
	East Asian	1	1	
	Hispanic	3	4	
Image quality	Mean	3.7256	3.8235	t (158) =-1.491, p =0.138
	SD	0.41429	0.41603	
How much teeth are showing?	None	18	38	
	A little	32	18	
	A lot	30	24	
	Mean	1.15	0.83	t (158) =2.512, p = 0. 013
	SD	0.765	0.868	

Makeup?	None	53	48	
	A little	27	32	
	Mean	0.34	0.40	t (158) =--0.816, p = 0.416
	SD	0.4576	0.493	
Emotion?	Neutral	17	23	x^2 (1) =1.20, p =0.273
	Happiness	63	57	
Facial hair?	None	73	50	
	A little	7	19	
	A lot	0	11	
	Mean	0.9	0.51	t (158) =-4.858, p <0.01
	SD	0.284	0.729	
Attractive	Mean	2.7409	2.7461	t (158) =-0.060，p = 0. 953
	SD	0.45786	0.64163	
Common?	Mean	3.2326	2.8334	t (158) = 8.151, p <0.01
	SD	0.29872	0.32064	
How much emotion is in this face?	Mean	3.1146	3.1668	t (158) =-0.464，p = 0.643
	SD	0.68326	0.73630	
Friendly	Mean	3.5471	3.4754	t (158) =-0. 757，p = 0.450
	SD	0.54510	0.64974	
Face direction?	At you	80	80	Na
Catch question	yes	80	80	Na
Eye’s direction?	At you	80	80	Na
Is this person famous?	No	80	80	Na

Note. LOW = low Memorability group, HIGH = high Memorability group, SD = standard deviation,

How memorable is this face? ： 1 (forgettable) - 5 (memorable)

Face's Age：1 = < 20 years、2 = 20 - 30 years、3 = 30 - 45 years、4 = 45 - 60 years、5 = 60+ years

Race ： races were selected based on common demographics of Amazon Mechanical Turk.

Image quality? ： 1 (poor) - 5 (very good)

Attractive ： 1 (unattractive) - 5 (attractive)

Common? ： 1 (uncommon) - 5 (common)

How much emotion is in this face? ： 1 (little) - 5 (a lot)

friendly ： 1 (very unfriendly - 5 (very friendly)

Table S2 Hit Rate (HR), False Alarm Rate (FAR), and demographic information for 58 high memorability images and 58 low memorability images. Statistics present independent t-test or x^2 test values investigating the group differences.

Attributes		LOW (n = 58)	HIGH (n =58)	Statistics
Hit Rate (HR)	Mean	0.346	0.667	t (114) = 28.530, p <0.0001
	SD	0.064	0.056	
Memorable	Mean	2.762	3.298	t (114) = 10.136, p <0.0001
	SD	0.237	0.326	
False Alarm Rate (FAR)	Mean	0.121	0.134	t (114) =1.187, p = 0.238
	SD	0.058	0.059	
Age	Mean	3.338	3.308	t(114) =0.182 ， p =0.856
	SD	0.794	0.925	
Gender	Female	30	32	x^2 (1) = 0.139， p =0.710
	Male	28	26	
Race	South Asian	0	1	x^2 (3) = 1.587， p =0.662
	Black	53	50	
	East Asian	3	5	
	Middle Eastern	2	2	
Image quality	Mean	3.741	3.788	t (114) =0.607， p =0.545
	SD	0.393	0.449	
How much teeth are showing?	None	12	27	
	A little	20	12	
	A lot	26	19	
	Mean	1.24	0.86	t (114) =2.446， p = 0. 016

	SD	0.779	0.888	
Makeup?	None	37	36	
	A little	21	22	
	Mean	0.36	0.38	t (114) =0.191， p = 0.849
	SD	0.485	0.489	
Emotion?	Neutral	11	17	x^2 (1) =1.695， p =0.193
	Happiness	47	41	
Facial hair?	None	56	36	
	A little	2	16	
	A lot	0	6	
	Mean	0.03	0.48	t (114) =4.835， p <0.001
	SD	0.184	0.682	
Attractive	Mean	2.723	2.680	t (114) =0.438， p = 0. 662
	SD	0.419	0.625	
Common?	Mean	3.219	2.846	t (114) = 6.697， p <0.001
	SD	0.294	0.305	
How much emotion is in this face?	Mean	3.150	3.140	t (114) =0.078， p = 0.938
	SD	0.676	0.711	
Friendly	Mean	3.575	3.429	t (114) =1.278， p = 0.204
	SD	0.550	0.678	
Face direction?	At you	58	58	Na
Catch question	yes	58	58	Na
Eye’s direction?	At you	58	58	Na
Is this person famous?	No	58	58	Na

Note. LOW = low Memorability group, HIGH = high Memorability group, SD = standard deviation,

How memorable is this face? ： 1 (forgettable) - 5 (memorable)

Face's Age：1 = < 20 years、2 = 20 - 30 years、3 = 30 - 45 years、4 = 45 - 60 years、5 = 60+ years

Race ： races were selected based on common demographics of Amazon Mechanical Turk.

Image quality? ： 1 (poor) - 5 (very good)

Attractive ： 1 (unattractive) - 5 (attractive)

Common? ： 1 (uncommon) - 5 (common)

How much emotion is in this face? ： 1 (little) - 5 (a lot)

friendly：1 (very unfriendly - 5 (very friendly)

The images utilized in the figures throughout our article have been carefully selected from a database of publication-friendly images. These images are solely intended for illustrative purposes and do not correspond to the actual visuals images employed in our experiments. The corresponding copyright information for these images in the figures is provided in the following table:

Pictures	Filename	URL	CopyrightOwner
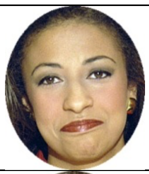	494043893_511442d9ca_z.jpg	http://www.flickr.com/photos/publicresourceorg/494043893/	Carl Malamud
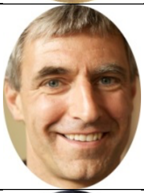	3617997999_4505a58d9c_o.jpg	http://www.flickr.com/photos/87553285@N00/3617997999/	Greg Niemeyer
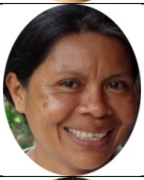	4451440734_0c5de7019d_o.jpg	http://www.flickr.com/photos/lonqueta/4451440734/	Lon & Queta
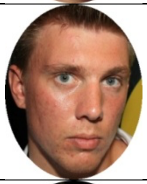	5045855615_f0419390bf_b.jpg	http://www.flickr.com/photos/swedenexpo/5045855615/	Tobias Andersson
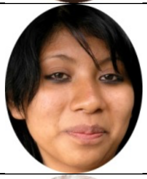	4732984233_0fb9c1789d_o.jpg	http://www.flickr.com/photos/lonqueta/4732984233/	Lon & Queta
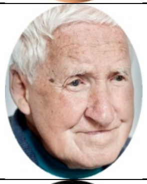	3973591816_12cdbd39ce_z.jpg	http://www.flickr.com/photos/levycarneiro/3973591816/	Levy Carneiro Jr
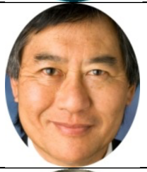	4900851395_1287eea7b5_o.jpg	http://www.flickr.com/photos/umdnews/4900851395/	Merrill College of Journalism
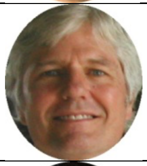	John_9-07.jpg	http://cervisa.com/	John Coate
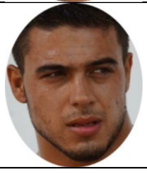	9251674623_8644f3b086_b.jpg	http://www.flickr.com/photos/charlesfred/9251674623/	Charles Roffey

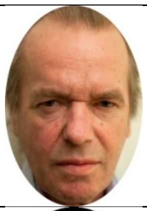	Martin_Amis_2012_by_Maximilian_Schoenherr.jpg	http://commons.wikimedia.org/wiki/File:Martin_Amis_2012_by_Maximilian_Schoenherr.jpg	Maximilian Schoenherr
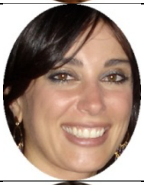	Nadine_Labaki.jpg	http://commons.wikimedia.org/wiki/File:Nadine_Labaki.jpg	Omernos
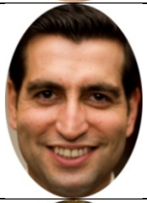	6334716732_3bfe43ba2c_o.jpg	http://www.flickr.com/photos/gregpc/6334716732/	Greg Peverill-Conti
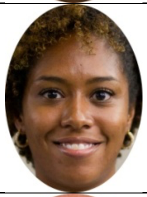	6147167672_e3bbdc52d2_o.jpg	http://www.flickr.com/photos/gregpc/6147167672/	Greg Peverill-Conti
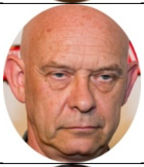	5728980527_c67e4e236d_o.jpg	http://www.flickr.com/photos/ianaberle/5728980527/	Ian Aberle
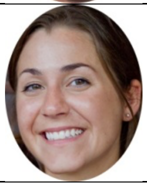	6153513558_044efa45d2_o.jpg	http://www.flickr.com/photos/gregpc/6153513558/	Greg Peverill-Conti

References

Bainbridge, W. A. (2019). Memorability: How what we see influences what we remember. In K. D. Federmeier & D. M. Beck (Eds.), *Psychology of Learning and Motivation: Advances in Research and Theory* (Vol. 70, pp. 1–27). Elsevier Inc. https://doi.org/10.1016/bs.plm.2019.02.001
